# Endosymbiosis in trypanosomatid protozoa: the bacterium division is controlled during the host cell cycle

**DOI:** 10.3389/fmicb.2015.00520

**Published:** 2015-06-02

**Authors:** Carolina M. C. Catta-Preta, Felipe L. Brum, Camila C. da Silva, Aline A. Zuma, Maria C. Elias, Wanderley de Souza, Sergio Schenkman, Maria Cristina M. Motta

**Affiliations:** ^1^Laboratório de Ultraestrutura Celular Hertha Meyer, Instituto de Biofísica Carlos Chagas Filho, Universidade Federal do Rio de Janeiro, Rio de Janeiro, Brazil; ^2^Instituto Butantan, São Paulo, Brazil; ^3^Center of Toxins, Immunology and Cell Signaling, São Paulo, Brazil; ^4^Instituto Nacional de Metrologia, Qualidade e Tecnologia, Xerém, Rio de Janeiro, Brazil; ^5^Escola Paulista de Medicina, Universidade Federal de São Paulo, São Paulo, Brazil

**Keywords:** symbiosis, cell evolution, trypanosomatid protozoa, cell cycle, division control

## Abstract

Mutualism is defined as a beneficial relationship for the associated partners and usually assumes that the symbiont number is controlled. Some trypanosomatid protozoa co-evolve with a bacterial symbiont that divides in coordination with the host in a way that results in its equal distribution between daughter cells. The mechanism that controls this synchrony is largely unknown, and its comprehension might provide clues to understand how eukaryotic cells evolved when acquiring symbionts that later became organelles. Here, we approached this question by studying the effects of inhibitors that affect the host exclusively in two symbiont-bearing trypanosomatids, *Strigomonas culicis* and *Angomonas deanei*. We found that inhibiting host protein synthesis using cycloheximide or host DNA replication using aphidicolin did not affect the duplication of bacterial DNA. Although the bacteria had autonomy to duplicate their DNA when host protein synthesis was blocked by cycloheximide, they could not complete cytokinesis. Aphidicolin promoted the inhibition of the trypanosomatid cell cycle in the G1/S phase, leading to symbiont filamentation in *S. culicis* but not in *A. deanei*. Treatment with camptothecin blocked the host protozoa cell cycle in the G2 phase and induced the formation of filamentous symbionts in both species. Oryzalin, which affects host microtubule polymerization, blocked trypanosomatid mitosis and abrogated symbiont division. Our results indicate that host factors produced during the cell division cycle are essential for symbiont segregation and may control the bacterial cell number.

## Introduction

Symbiotic relationships between unicellular organisms, such as protozoa and bacteria, constitute interesting models for the investigation of organelle division and segregation during the cell cycle. Obligatory symbiosis usually involves control over the number of symbionts and the establishment of mechanisms to ensure that the cell will inherit at least one symbiont during its division.

Trypanosomatids are flagellated protozoa that carry a single copy of essential structures, such as the basal body, flagellum, nucleus and kinetoplast, an enlarged portion of the mitochondrion that contains circular and interlocked DNA (kDNA). Thus, such protozoa constitute interesting models to investigate the mechanisms that orchestrate the equal distribution of structures between daughter cells (Steinert and Van Assel, 1967; Crosgrove and Skeen, 1970; Woodward and Gull, 1990). Seven trypanosomatid species co-evolve with a single obligate bacterium that divides in synchronization with the host cell, thus providing an opportunity to study cell cycle regulation and the evolution of symbiotic associations (Motta et al., 2010; Brum et al., 2014). Recently, symbiont-bearing trypanosomatids were reclassified into three genera: *Angomonas*, *Strigomonas*, and *Kentomonas* (Teixeira et al., 2011; Votýpka et al., 2014).

In other models where protozoa and prokaryotes co-exist in symbiosis, usually dozens to hundreds of symbionts are present in the host cytoplasm, as observed in the free-living protozoa *Amoeba proteus* (Jeon, 2006). In such models, somehow symbionts are protected from digestion and contribute to the host metabolism (Ahn and Jeon, 1979). However, the mechanisms used by hosts to control the symbiont number are still poorly understood (Nowack and Melkonian, 2010). In trypanosomatids, the symbiont number and division control are tightly regulated; thus, each daughter cell carries only one bacterium at the end of the cell cycle (Motta et al., 2010; Brum et al., 2014).

Endosymbiosis in trypanosomatids results from a monophyletic event, and the bacterial genome is greatly reduced compared with the probable ancestral β-proteobacterium, within the Alcaligenacea family (Alves et al., 2011). Genes related to division and cell wall synthesis are lost in trypanosomatid symbionts, whereas those involved in housekeeping functions, such as DNA synthesis and repair, are maintained (Motta et al., 2013). The symbiotic bacteria also preserved genes which code enzymes that complete essential metabolic pathways of the host trypanosomatid, such as heme, amino acids and vitamin production (Alves et al., 2011, 2013; Klein et al., 2013). It means that symbiont-harboring trypanosomatids present low nutritional requirements when compared to other species of the family (reviewed, by Motta, 2010).

Although genomic similarity is observed among the symbionts of different trypanosomatid species, recent phylogenetic analyses have indicated an evolutionary divergence among bacteria from distinct genera (Alves et al., 2011). Indeed, our previous studies have shown that each symbiont exhibits distinct forms and positions during the host protozoan cell cycle. Nevertheless, in both species, the bacterium divides just before the segregation of the protozoan kinetoplast and nucleus (Motta et al., 2010; Brum et al., 2014).

To further understand how symbiont segregation is coordinated with the protozoan division, herein, we investigated the effects of inhibitors that specifically affect the host cell cycle in distinct phases. Our results provide evidence that symbiont segregation, but not DNA duplication, is dependent on the progression of the protozoan cell division cycle, indicating that the host trypanosomatid exerts tight control over the bacterial cell number. Furthermore, inhibitors differently affected symbiont division in *A. deanei* and *S. culicis*, showing that partners co-evolve in distinct ways in each species.

## Materials and Methods

### Protozoa Growth

The *Angomonas deanei* normal strain (ATCC 30255), *Angomonas deanei* aposymbiotic strain (ATCC 044), *Strigomonas culicis* normal strain (ATCC 30268), and *Strigomonas culicis* aposymbiotic strain (ATCC 30257) were grown at 28°C in Warren’s culture medium (Warren, 1960) supplemented with 10% fetal bovine serum. Aposymbiotic strains were artificially generated after antibiotic treatment and were maintained in the laboratory in supplemented medium (Chang, 1974; Mundim and Roitman, 1975). Experiments were performed using cells cultivated for 24 h, which corresponded to the exponential growth phase for both species.

### Inhibitor Treatments

Cycloheximide, a eukaryotic protein synthesis inhibitor, was used at 1, 5, 10, and 25 μM; m-divi1, an inhibitor of mitochondrial dynamin, was employed at 25, 50, 100, and 200 μM; aphidicolin, an inhibitor of eukaryotic DNA polymerase, was used at 30, 60, and 90 μM; camptothecin, an inhibitor of eukaryote topoisomerase I that induces DNA breaks, was employed at 1, 5, 10, 50 μM; and oryzalin, a microtubule depolymerization inducer known to block mitosis, was used at 1, 5, 25, and 50 μM. The actions of these inhibitors are shown in Table 1. All of the drugs were obtained from Sigma Aldrich (St. Louis, MO, USA) except m-divi1, which was purchased from Millipore (Darmstadt, Germany). The compounds were dissolved according to the manufacturers’ instructions, and controls of the diluents were prepared when necessary. The cells were inoculated at a concentration of 1 × 10^6^ mL^–1^ in culture medium; after 12 h, the indicated drug concentrations were added. Next, the cells were collected every 12 h until 60 h and then were processed as described above. Reversibility assays were performed after 24 h and 48 h of treatment, and then the cells were centrifuged at 2,000 *g* for 10 min to remove the inhibitors, washed twice with phosphate-buffered saline (PBS, pH 7.2) and resuspended in fresh medium containing 10% fetal bovine serum.

**TABLE 1:** **Inhibitors effects**.

**Inhibitors**	**Effect**
Cycloheximide	Eukaryotic protein synthesis inhibition
m-divi1	Dynamin related protein inhibition
Aphidicolin	G1/S-phase arrest
Camptothecin	G2/M-phase arrest
Oryzalin	Mitosis impairment

### Viability Assays

An aqueous solution of the MTS [(3-(4,5-dimetiltiazol-2-il)-5-(3-carboximetoxifenil)-2-(4-sulfofenil)-2H-tetrazolium)] CellTiter MTS Reagent (Promega, Woods Hollow, USA) was prepared in PBS to a final concentration of 2 mg mL^–1^. The solution was protected from light and shaken for 15 min, or until the MTS was completely dissolved. The pH of the solution was adjusted to 6.0–6.5 with 1 N HCl, and then the solution was sterilized by filtration through a 0.2-μM filter, and stored at –20°C in aliquots. The phenazine methosulfate (PMS) stock solution was prepared in PBS to a final concentration of 3 mM, filter sterilized, aliquoted and stored at –20°C, protected from light. To perform the MTS/PMS assay, 50 μL of PMS stock solution was added to 1 mL of MTS stock solution immediately before use. Then, 20 μL of the MTS/PMS mixture was added to each well containing 10^6^ cells in 100 μL of PBS with 4 mM glucose, resulting in a final quantity of 40 μg (333 μg mL^–1^) of MTS and 0.92 μg (25 μM) of PMS per well (Promega, Technical Bulletin). The 96-well plates were incubated at 28°C for 4 h, and the absorbances were read at 490 nm using a microplate reader (Spectra Max Molecular Devices M2e). Negative controls consisted of cells fixed with 0.4% formaldehyde for 10 min at room temperature.

### Immunofluorescence Assays

The evaluation of protozoa cellular patterns and symbiont forms was performed as follows. Protozoa were washed in PBS and fixed with freshly prepared 1% formaldehyde in PBS for 1 h. After fixation, the cells were deposited on poly-L-lysine-coated microscope coverslips (20 × 20 mm) and permeabilized with 2% Nonidet P-40 (NP-40) diluted in PBS for 30 min. The slides were incubated in blocking solution containing 1.5% bovine serum albumin (BSA), 0.5% teleostean gelatin (Sigma Aldrich), and 0.02% Tween 20 in PBS. Next, the slides were incubated for 1 h with antibody against the symbiont porin (Andrade et al., 2011) diluted 1:5 in blocking solution. After the incubation with the primary antibody, the cells were washed and incubated for 45 min with Alexa 488-conjugated anti-mouse IgG (Molecular Probes, USA) diluted in blocking solution to final concentration of 3 μg mL^–1^. Samples incubated with pre-immune sera or not incubated with the primary antibodies were used as negative controls. The slides were mounted using the anti-fade reagent ProLong Gold containing 5 μg mL^–1^ of DAPI (4′,6-diamidino-2-phenylindole), (Molecular Probes). Serial image stacks (0.2-μm Z-increment) were collected at 100 × (oil immersion 1.4 NA) on a motorized Olympus BX microscope equipped with differential interference contrast optics and an Orca R2 camera (Hamamatsu, Japan). All of the images were collected using Cell^^^M software (Olympus, USA), and the fluorescence images were deconvolved using blind deconvolution and AutoQuant 2.2 software (Media Cybernetics, USA).

### Cell Cycle Analysis

**FIGURE 1:**
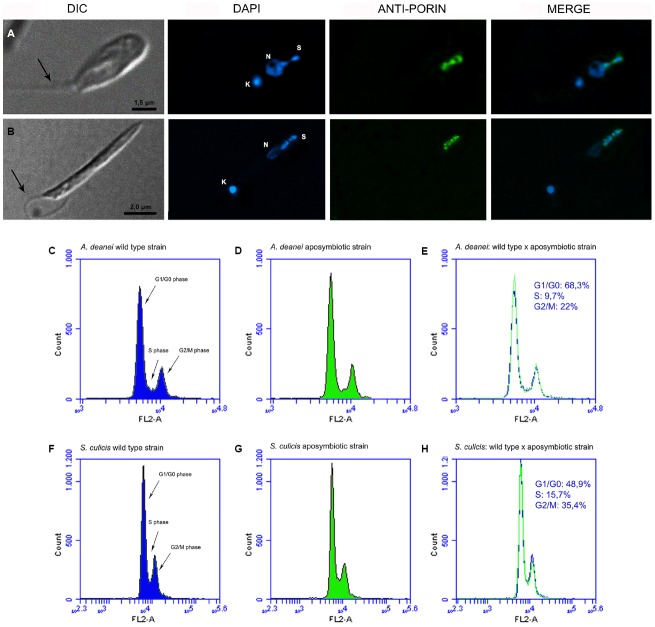
**Cell cycle in control cells.** The top panels show optical microscopy images of *A. deanei* **(A)** and *S. culicis* **(B)** observed by differential interference contrast (DIC) and fluorescence microscopy after staining with DAPI and anti-porin, a specific antibody that labels the symbiont. The black arrow indicates the flagellum. The bottom panels show the flow cytometry histograms of wild-type, aposymbiotic strains and the merged analyses of *A. deanei* **(C–E)** and *S. culicis* **(F–H)**. The sizes of the scale bars are indicated in each figure.

After the indicated treatments, the cells were washed in PBS and fixed with 0.25% freshly prepared formaldehyde diluted in PBS for 30 min. Then, the cells were washed once in PBS and fixed again for another 30 min in 1 mL of cold 70% ethanol added dropwise to cells while vortexing to avoid clumping. After harvesting and washing, the cells were incubated with 25 μg mL^–1^ RNase A and 5 μg mL^–1^ propidium iodide (PI) for 30 min at 37°C to stain the DNA. Unstained samples were used as the control. Analyses were performed promptly using a BD Accuri C6 system (BD Biosciences, USA) acquiring at least 10,000 events. In all of the experiments, the aposymbiotic strain of both species was used as a control to guarantee that the symbiont DNA does not affect the analysis. In the G1 phase, DNA is not duplicated; thus, the G1 phase cells correspond to the fluorescent peak at the left. The G2/M-phase population contains cells with duplicated DNA and corresponds to the peak at the right. Cells in S phase are represented between peaks (Figures 1C,F).

The cell cycle was also evaluated by fluorescence microscopy after DAPI staining, with the cellular patterns determined by counting DNA-containing structures (nuclei, kinetoplasts and symbionts) as well as the number of flagella. Symbiont division was evaluated based on its form as described previously (Motta et al., 2010; Brum et al., 2014). Non-treated cells were used as the control. Analyses were based on counts of 1,000 cells.

### Transmission Electron Microscopy

Transmission electron microscopy (TEM) analyses were performed in control and treated protozoa to check the integrity of cellular structures, particularly in the symbiont. Protozoa were washed twice in PBS and fixed in 2.5% glutaraldehyde in 0.1 M cacodylate buffer, pH 7.2, for 1 h. After washing again in 0.1 M cacodylate buffer, pH 7.2, cells where postfixed for 1 h in 1% osmium tetroxide containing 0.8% potassium ferrocyanide, 5 mM CaCl_2_ in 0.1 M cacodylate buffer. After postfixation, cells were washed, dehydrated in a series of increasing acetone concentrations and embedded in Epon—first as a mixture of Epon and acetone (1:1) and then as pure Epon (Bozzola and Russel, 1998). Ultrathin sections were obtained using an Ultracut Reichert Ultramicrotome and mounted on 400-mesh copper grids, and then the sections were stained with uranyl acetate and lead citrate. Samples were analyzed using a Zeiss 900 or 902 transmission electron microscope.

## Results

### Cell Cycle in Control Cells

We initially confirmed the symbiont proximity to the nucleus in control cells by immunofluorescence analysis (Figures 1A,B). The close association between the symbiont and the protozoan nucleus, as well as to the endoplasmic reticulum, was also evident by TEM images of *A. deanei* and *S. culicis* (Supplementary Figures S 1A and S 2A). Previously, immunofluorescence analysis showed that most protozoa presented a cellular pattern containing single copies of essential structures, such as the flagellum, nucleus, and kinetoplast, as well as a symbiont, which was present in a constricted form containing duplicated DNA (1N1K1F1S∞). Flow cytometry of exponentially growing cells of wild-type and aposymbiotic strains revealed that the presence of the symbiotic bacterial DNA did not influence the fluorescence histogram peaks (Figures 1E,H). In both species, most of the protozoa were in the G1 phase, corresponding to 68.3% of the total cells in *A. deanei* (Figures 1C–E) and 48.9% in *S. culicis* (Figures 1F–H). In *A. deanei*, the S- and G2-phase populations constituted 9.7 and 22% of the total population, respectively (Figures 1C–E), while 15.7% of *S. culicis* cells were in S phase, and 35.4% were in G2 phase (Figures 1F–H).

### Protein Synthesis Arrest Induced by Cycloheximide Prevents Symbiont Division

Cycloheximide promoted strong inhibition of the proliferation of *A. deanei* and *S. culicis* at concentrations equal to or greater than 1 μM in the first 12 h of treatment. Higher concentrations, such as 10 and 50 μM, completely abolished cell growth. The proliferation was restored when cells treated for 48 h were washed and cultivated in fresh medium (S 3A–B).

**FIGURE 2:**
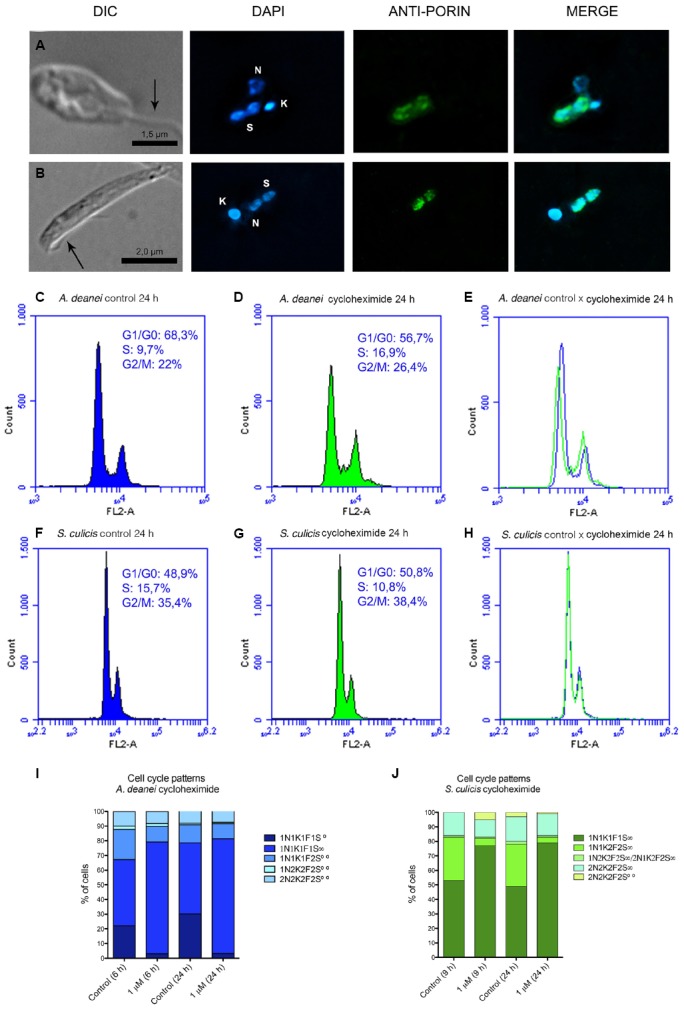
**Cycloheximide affects endosymbiont division.** The top panels show optical micrographs of *A. deanei* **(A)** and *S. culicis* **(B)** treated for 24 h with 1 μM cycloheximide. The pictures correspond to differential interference contrast (DIC), DAPI, anti-porin staining and the merged images. The black arrows indicate the flagellum. The sizes of the scale bars are indicated in each figure. The middle panel shows flow cytometry histograms of DNA labeling in control cells **(C,F)** and protozoa treated for 24 h with 1 μM cycloheximide **(D,G)**, together with the merged histograms **(E,H)**. The numbers inside the histograms represent the mean G1, S and G2 percentages ± SEM. The bottom panel represents the cell pattern distribution generated by counting DNA-containing structures of *A. deanei* **(I)** and *S. culicis* **(J)** after the indicated treatments. F, flagellum; K, kinetoplast; N, nucleus; S, symbiont. S∞, a single symbiont in rod shape per cell, S∞—a single symbiont in constriction (dividing format) per cell, S∞∞—two symbionts in rod shape per cell, S∞∞∞— filamentous symbiont.

Cells treated with 1 μM cycloheximide for 24 h and observed by immunofluorescence mostly presented one flagellum, one kinetoplast, one nucleus and a single symbiont in a constricted form (1N1K1F1S∞; Figures 2A,B). Flow cytometric analyses revealed that the cell cycle was not arrested in a specific phase after cycloheximide treatment, but instead resulted in protozoa arrested in different phases. *A. deanei* presented a small increase in the S- and G2-phase populations (Figures 2C–E), while no significant change was found in the case of *S. culicis* (Figures 2F–H). Cycloheximide treatment decreased the number of protozoa containing duplicated symbionts and increased the percentage of cells containing only one constricted bacterium (1N1K1F1S∞) to approximately 80% in *A. deanei* and *S. culicis* after 6 and 9 h, respectively. Cells containing duplicated structures (2N2K2F2S) did not show modifications in their percentages (Figures 2I,J). TEM of both species treated with 1 μM cycloheximide for 24 h mainly showed symbionts with a preserved envelope and the classical halter shape, indicating that its genetic material was duplicated. Location of the symbiont in close proximity to the endoplasmic reticulum or nucleus was less frequent than in control cells (S 1A–B and S 2A–B). Together, these results indicate that cycloheximide did not prevent symbiont DNA replication but abrogated its DNA segregation, thus reducing the number of cells containing two bacteria.

### M-divi1 Does Not Affect Symbiont Division

The effects of m-divi1 on the proliferation of both species were observed after 24 h of treatment. *A. deanei* appeared to be more sensitive to this inhibitor than *S. culicis*; thus, a significant reduction in cell growth was observed after treatment with 100 or 200 μM of the drug, respectively (S 5A,B). For all concentrations tested, the m-divi1 effect was reversible after 48 h of treatment and did not reduce viability (S 4, S 5A,B). Cell cycle arrest was not observed in cytometry analyses when the protozoa were treated with concentrations that inhibit proliferation (S 5C–H). Immunofluorescence images showed symbionts during the division process (S 6A,B), and no differences in the cellular patterns were found in *A. deanei* or *S. culicis* treated with 100 μM or 200 μM m-divi1, respectively (S 6C,D). The protozoa did not present ultrastructural alterations as revealed by TEM analyses (S 6E,F). These results indicate that drp is not directly involved with symbiont segregation and does not seem to have effects on trypanosomatid mitochondrion division or cell cycle progression.

### Aphidicolin Promotes Protozoa DNA Synthesis Arrest and Induces Symbiont Filamentation in *S. culicis*

Next, we examined the bacterial symbiont division when the host protozoa cell cycle was blocked using aphidicolin, an inhibitor of eukaryotic DNA polymerase. *A. deanei* and *S. culicis* presented different sensitivities to aphidicolin in their growth. While 60 μM aphidicolin was required to stop *A. deanei* proliferation (S 3C) without interfering with cell viability (S 4), half of this dose (30 μM) caused the same effect in *S. culicis* (S 3D and S 4). In both species, the effect on proliferation was reversible after 48 h of treatment (S 3C,D).

**FIGURE 3:**
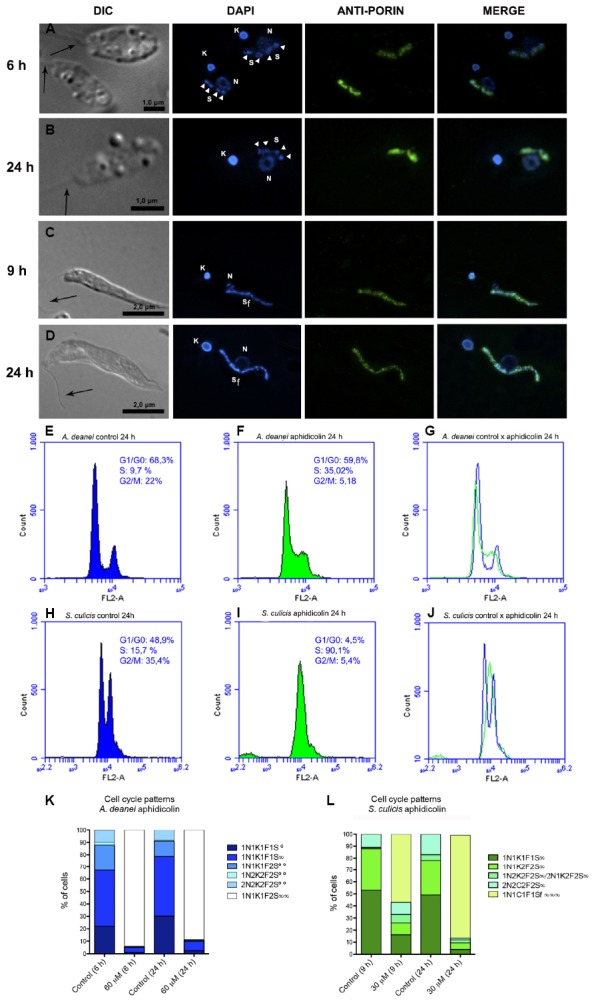
**Aphidicolin affects symbiont division in *A. deanei* and *S. culicis*.** The top panels show optical micrographs of *A. deanei* **(A,B)** and *S. culicis* **(C,D)** treated with 60 μM and 30 μM aphidicolin up to 24 h, respectively, and labeled with DAPI and anti-porin. The black arrows indicate the flagellum, and the white arrowheads indicate the symbiont’s nucleoids. The sizes of the scale bars are indicated in each figure. The middle panels show flow cytometry histograms of control *A. deanei* **(E)** and *S. culicis* **(H)**, or cells treated for 24 h with 60 μM **(F)** or 30 μM **(I)** aphidicolin, respectively. Merged histograms are represented on the right **(G,J)**. The bottom panel represents the cell pattern distribution generated by counting DNA-containing structures of *A. deanei* **(K)** and *S. culicis* **(L)** after the indicated treatments. F, flagellum; K, kinetoplast; N, nucleus; S, symbiont. S∞—a single symbiont in rod shape per cell, S∞—a single symbiont in constriction (dividing format) per cell, S∞∞—two symbionts in rod shape per cell, S∞∞—filamentous symbiont.

Immunofluorescence analyses revealed that aphidicolin treatment affects the symbiont morphology (Figures 3A–D). Approximately 90% of *A. deanei* cells had two symbionts, each one with two nucleoids after treatment for 6 and 24 h, and only one nucleus and kinetoplast (1N1K1F1S∞∞; Figure 3A,B,K). Even when cells were observed after 48 h of cultivation in the presence of aphidicolin, the symbiont number was maintained, and filamentous bacteria were not observed. Importantly, cells containing more than one nucleus and one kinetoplast were rare. By contrast, 30 μM aphidicolin promoted the filamentation of *S. culicis* symbionts after 9 h of treatment, with 57% of cells presenting this pattern. After 24 h of treatment, longer filaments were observed in almost 90% of the population (Figure 3C,D,L). Even after 120 h, we observed enlarged filaments (data not shown). According to flow cytometry analyses, *A. deanei* cultivated with 60 μM for 24 h was partially arrested in S phase (35%), with a small decrease in the percentage (59.8%) of cells remaining in G1 (Figures 3E–G). By contrast, most *S. culicis* cells were arrested in S phase (90.1%) after 24 h of treatment with 30 μM aphidicolin (Figures 3H–J).

By TEM analyses, we observed *A. deanei* containing two symbionts in a constricted form; in some sections, we observed the bacteria in association with the nucleus (S 1C,D). When *S. culicis* was visualized by TEM, we noticed several symbiont profiles, which are compatible with the presence of a long filamentous bacterium (S 2C). Taken together, these results indicate that symbionts of both species can duplicate their DNA independently of the protozoan. However, the host cell cycle progression is necessary for bacterial cytokinesis.

### Camptothecin Blocks the Host Cell Cycle in the G2 Phase and Promotes Symbiont Filamentation in both Species

**FIGURE 4:**
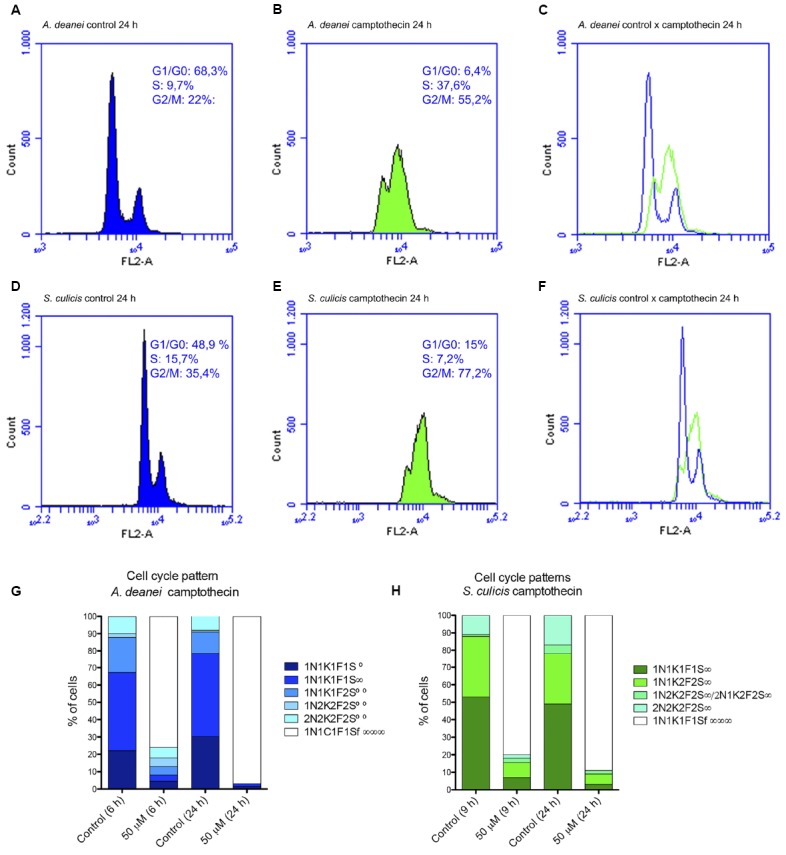
**The top panel shows flow cytometry histograms of control *A. deanei* (A) and *S. culicis* (D), or cells treated for 24 h with 10 μM camptothecin (B,E).** Merged histograms are represented on the right **(C,F)**. The bottom panel represents the cell pattern distribution generated by counting DNA-containing structures of *A. deanei* **(G)** and *S. culicis* **(H)** after the indicated treatments. F, flagellum; K, kinetoplast; N, nucleus; S, symbiont. S∞—a single symbiont in rod shape per cell, S∞—a single symbiont in constriction (dividing format) per cell, S∞∞—two symbionts in rod shape per cell, S∞∞∞—filamentous symbiont.

**FIGURE 5:**
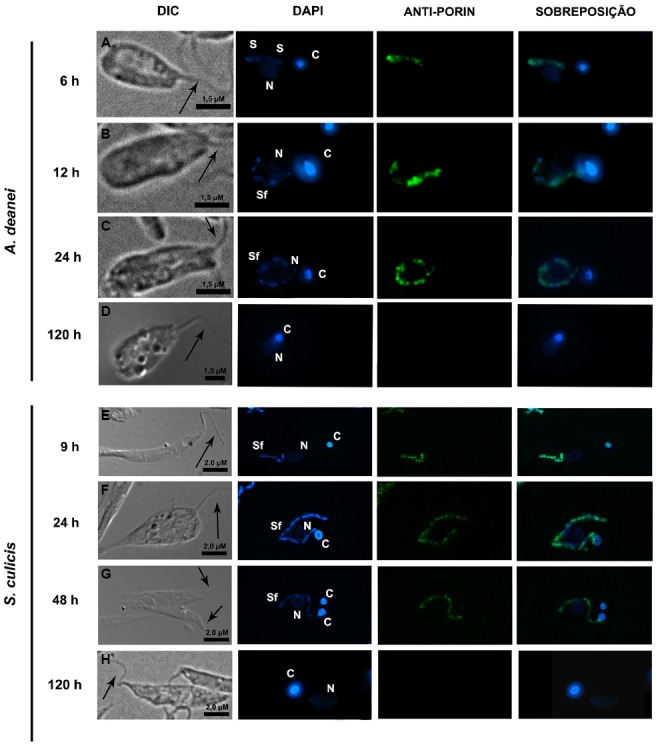
**Camptothecin promotes the symbiont filamentation in *A. deanei* and *S. culicis*.** The panels show optical micrographs of *A. deanei* **(A–D)** and *S. culicis* **(E–H)** treated up to 120 h with 10 μM camptothecin and labeled with DAPI and anti-porin. The black arrow indicates the flagellum. The sizes of the scale bars are indicated in each figure.

Proliferation of both protozoan species was affected by 10 μM camptothecin (S 3E,F). The effect on proliferation was reversible in cells treated with 5 and 10 μM but not with 50 μM. Viability assays showed that cell viability was affected only after 48 h of treatment with 10 μM camptothecin (S 3K,L). Therefore, we treated both symbiont-bearing species with 10 μM camptothecin for up to 120 h. Cytometry analysis showed that the inhibitor blocked the cell cycle in the G2/M phase after 24 h. In *A. deanei*, 55.2% of cells were arrested in this phase; however, in *S. culicis*, this percentage was 77.2% (Figures 4A–F). Importantly, camptothecin treatment promoted symbiont filamentation in both species (Figures 5A–H). The counting of DNA-containing structures and flagella showed that, after treatment with 10 μM camptothecin for 24 h, 97 and 89% of *A. deanei* and *S. culicis* cells, respectively, exhibited 1 nucleus, 1 kinetoplast, 1 flagellum, and 1 filamentous symbiont (1N1K1F1Sf∞∞∞; Figures 4G–H). In *S. culicis*, a small portion of cells exhibited a duplicated kinetoplast and two flagella, indicating the occurrence of cytokinesis (Figure 5G). With longer camptothecin treatment, such as 120 h, we noticed the appearance of aposymbiotic cells (Figures 5D,H). After 144 h of camptothecin treatment, 60% of *A. deanei* cells no longer presented the symbiont, and the percentage was equivalent to 35% in *S. culicis* (data not shown). Filamentous symbionts were also visualized by TEM, which showed constrictions in the elongated form of the *A. deanei* bacterium (S 1E,F). In *S. culicis*, the filamentous symbiont appeared to be shapeless, and constricted regions were not observed (S 2D). These results indicate that, although differences were seen in the forms of symbiont filaments observed in *A. deanei* and *S. culicis*, in both cases, symbiont division is not coordinated with the G2 phase of the nucleus.

### Mitosis Arrest by Oryzalin Prevents Symbiont Division in *S. culicis*

**FIGURE 6:**
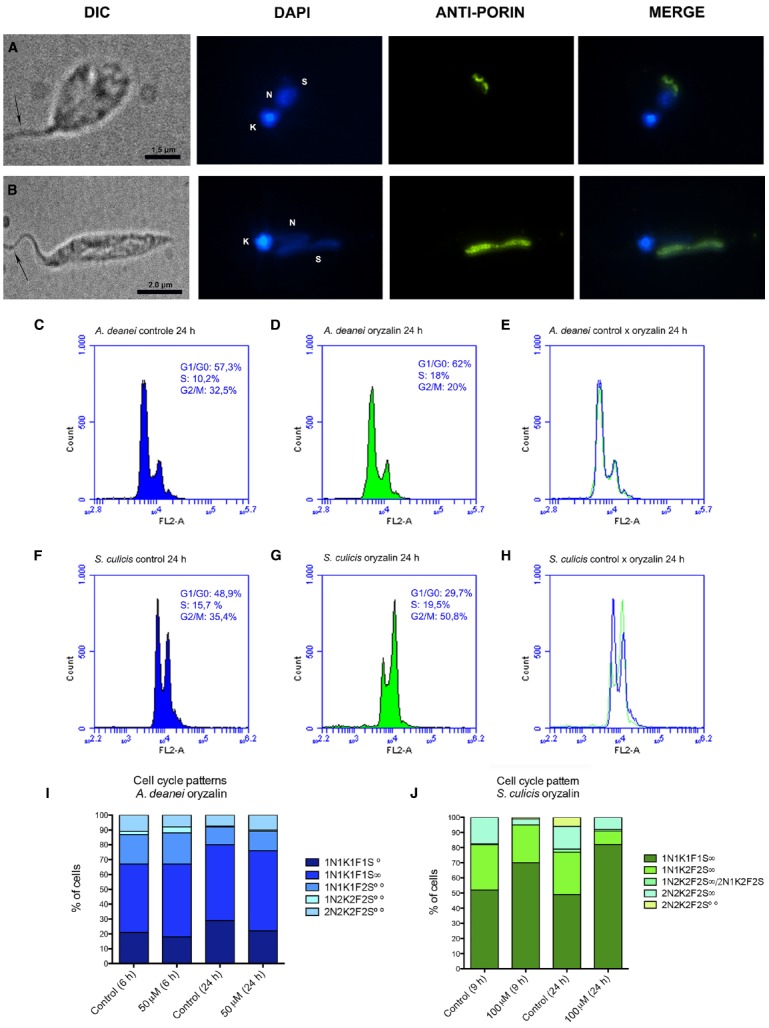
**Oryzalin treatment abrogates symbiont segregation in *S. culicis* but not in *A. deanei*.** The top panels show optical micrographs of *A. deanei* **(A)** and *S. culicis* **(B)** treated for 24 h with 50 μM oryzalin and labeled with DAPI and anti-porin. The black arrow indicates the flagellum. The sizes of the scale bars are indicated in each figure. The middle panels show flow cytometry histograms of control *A. deanei* **(C)** and *S. culicis* **(F)**, or cells treated for 24 h with 50 μM oryzalin **(D,G)**. Merged histograms are shown on the right **(E,H)**. The bottom panels show the cell pattern distribution generated by counting DNA-containing structures of *A. deanei* **(I)** and *S. culicis* **(J)** after the indicated treatments. F, flagellum; K, kinetoplast; N, nucleus; S, symbiont. S∞—a single symbiont in rod shape per cell, S∞—a single symbiont in constriction (dividing format) per cell, S∞∞—two symbionts in rod shape per cell.

Next, we asked whether the symbiont division occurred in the host protozoan cell cycle arrested in mitosis by oryzalin. The concentrations of the inhibitor that affected *A. deanei* and *S. culicis* proliferation after 12 h of treatment were distinct and corresponded to 50 and 100 μM, respectively (S 3I,J). In *A. deanei*, the proliferation was reverted after treatment with 50 μM (S 1I), but this concentration affected cell viability (S 4), indicating that it was not suitable for our tests. Conversely, in *S. culicis*, the effect of 50 μM oryzalin was reversible (S 3J) and did not affect cell viability (S 4). Flow cytometry analysis showed small modifications in the G1- and S-phase cell percentages for *A. deanei* (Figures 6C–E) were probably related to the loss in cell viability. Different concentrations of oryzalin did not promote alterations in the symbiont form and cellular patterns of *A. deanei* (Figures 6A,I). By contrast, this inhibitor affected the *S. culicis* cell cycle as demonstrated by flow cytometry analysis, which showed an increase in the cell number in the G2/M phase from 35.4 to 50.8% (Figures 6F–H). Importantly, the immunofluorescence data and cell cycle distribution pattern indicated that, when *S. culicis* was treated with oryzalin for 24 h, host mitosis did not occur and the symbiont did not divide, remaining mostly in the constricted form. The symbiont presented a more elongated shape but did not form filaments, as observed when the G1 and G2 phases were blocked by aphidicolin and camptothecin, respectively (Figures 6B). After 9 h and 24 h of treatment with 50 μM oryzalin, 70 and 82% of the cells in culture presented 1N1K1F1S∞, respectively (Figure 6J). The TEM analyses showed the maintenance of the symbiont and nucleus proximity, as well as the integrity of structures in *S. culicis* (S 2E,F) that included the formation of the mitotic spindle (S 3E,F). These results indicate that mitosis in *S. culicis* was blocked by oryzalin and that the symbiont probably coordinates its own cell cycle with that of the host nucleus.

## Discussion and Conclusion

One of the key events involved in the maintenance of a mutual benefit symbiosis is the control and regulation of the symbiont number inside the host cell. Here we showed that different types of eukaryotic inhibitors that cause growth arrest of the host protozoan prevented endosymbiont division. In some cases, bacterial DNA replication continues and generates filamentous structures, indicating that the control of symbiont division is established during host cell cycle progression. The *A. deanei* generation time is approximately 6 h, and that of *S. culicis* is 9 h, while symbiont replication occurs in 4 h. Therefore, it is reasonable to assume that the symbiont waits for a host cell signal to complete its cytokinesis (Motta et al., 2010; Brum et al., 2014). It is interesting to mention that in mutualistic associations between fungi and plants and between bacteria and plants, signaling molecules, as phosphoinositides, are directly involved in the establishment and maintenance of symbiotic relationships (Basu et al., 1999; Engstrom et al., 2002; Plett et al., 2011). Symbiotic population control and nodulation is well described in *Glicine max* and its symbiontic bacteria *Bradyrhizobium japonicum*, where the host controls nodulation by repressing the symbiont gene nodD2 (Jitacksorn and Sadowsky, 2008).

Genomic analyses have shown that symbionts, from *A. deanei* and *S. culicis*, present reduced genomes with similar sizes (≈830 kb). There is a significant loss of genes related to division, such as those from the *fts* family (filament temperature sensitive), and cell wall synthesis compared with free-living prokaryotes (Motta et al., 2013). Accordingly, ultrastructural analyses have shown a reduced peptidoglycan layer and the lack of a septum and a Z-ring (Soares and De Souza, 1988; Motta et al., 1997, 2004), suggesting that the symbiont division would depend on the host factors. This idea is also supported by our results showing that blocking protozoan protein synthesis with cycloheximide prevented symbiont cytokinesis.

Dynamins found in eukaryotic cells control the balance between the fusion and fission of organelles with symbiotic origin, particularly drp, which is the main protein responsible for mitochondrion division (Margolin, 2005). However, inhibition of mitochondrial dynamin by m-divi1 did not induce the blockade of symbiont segregation, indicating that another constriction system is responsible for this process. Alternatively, m-divi1 did not act as a dynamin inhibitor in symbionts containing trypanosomatids.

In the present study, inhibitors differentially affected *A. deanei* and *S. culicis*, suggesting that a complex process involving factors and signals controls the coordinated division and inheritance of the symbiont in each host species. For example, treatment with aphidicolin, which blocks the cell cycle in the G1/S phase, only promoted filamentation in symbionts of *S. culicis*. By contrast, cell cycle arrest in the G2/M phase promoted by camptothecin generated filamentous symbionts in both species. Moreover, treatment with this compound led to the appearance of cured protozoa as result of symbiont lysis after filament disruption. Treatment of *A. deanei* with β-lactam antibiotics generated filamentous symbionts and promoted bacterium lysis (Motta et al., 1997). In all cases, the symbiont filamentation is probably related to the continuous synthesis of the prokaryote DNA, whereas bacterial fission depends on factors produced by the host. In this sense, bacteria lacking division proteins also form filaments in the absence of the septum, because the genetic material is normally replicated (Lutkenhaus and Addinall, 1997).

Treatment with oryzalin promoted cell cycle arrest in the G2/M phase, abrogating mitosis in *S. culicis* and abolishing symbiont division, indicating that bacterial segregation depends on nuclear mitosis and/or microtubule organization. It is important to note that, in contrast to the filamentation observed after treatment with other cell cycle inhibitors, oryzalin did not induce bacterium filamentation. This finding indicates that symbiont division, but not DNA replication, may be controlled during cell cycle progression through the S and G2 phases, at least in the case of *S. culicis*.

In trypanosomatids, cell cycle control has mainly been studied in *T. brucei* with the possibility of silencing gene expression by RNA interference. Studies with this protozoan have shown that mitosis impairment does not prevent the replication of other structures and cytokinesis, as demonstrated when cyclin 6, a protein involved in mitosis induction, is silenced in procyclic cells. By contrast, the bloodstream form of *T. brucei* perform several rounds of kinetoplast division but do not complete mitosis or cytokinesis (Hammarton et al., 2003; Mckean, 2003). *T. brucei* treatment with aphidicolin leads to asymmetric cytokinesis, generating cells without nuclei, also known as zooids (Ploubidou et al., 1999). Indeed, we observed that treatment with aphidicolin generated 2% of dyskinetoplastic cells in *A. deanei* and *S. culicis*, which exhibited one nucleus and one filamentous symbiont. The presence of these abnormal patterns suggests the lack of a checkpoint that prevents cytokinesis in the absence of mitosis, as observed in the *T. brucei* procyclic form (Hammarton et al., 2003).

**FIGURE 7:**
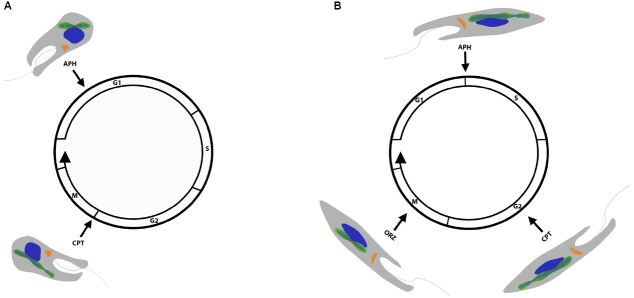
**Schematic representation showing the effect of inhibitors on the host protozoan cell cycle and on symbiont division.** Aphidicolin arrested the *A. deanei* **(A)** cell cycle in the G1/S phase, and the symbiont underwent DNA replication, generating a bacterium with four nucleoids; however, the cytokinesis was not completed. In *S. culicis* **(B)**, the same inhibitor blocked the host cell cycle in the S phase and induced symbiont filamentation, indicating different division control of the symbiont in each species. Camptothecin induced host cell cycle arrest in the G2 phase and symbiont filamentation in both species. Oryzalin only showed an effect on the *S. culicis* cell cycle, which was arrested in the M phase, thus impairing symbiont division.

In the current work, our results revealed that, in symbiont-bearing trypanosomatids, there is a close relationship between the cell cycle progression of the host and bacterial division, which is differentially regulated in each species. In *A. deanei*, the coordinated division between the prokaryote and host cell is established in the G1/S phase, whereas it occurs later in *S. culicis*, during the G2/M phase. Observations that lead to these conclusions are summarized in Table 2 and Figure 7. Such differences in division synchronicity may be related to the phylogenetic divergence that occurred during the co-evolution between trypanosomatids and their respective symbionts (Alves et al., 2011). This work reinforces the idea that mutualistic relationships, such as endosymbiosis in trypanosomatids, represent excellent models to better understand the division control of symbiont-derived organelles in eukaryotes.

**TABLE 2:** **Effects of the inhibitors on the host protozoa cell cycle and symbiont division**.

	**Cycloheximide**	**m-divi1**	**Aphidicolin**	**Camptothecin**	**Oryzalin**
***A. deanei***			G1/S-phase arrest		
	Inhibition of symbiont cytokinesis	Did not affect cell cycle progression or symbiont division	Maximum of 4 symbiont nucleoids per cell	G2/M-phase arrest Symbiont filamentation	
***S. culicis***			G1/S-phase arrest Symbiont filamentation		G2/M-phase arrest Inhibition of symbiont cytokinesis

### Conflict of Interest Statement

The authors declare that the research was conducted in the absence of any commercial or financial relationships that could be construed as a potential conflict of interest.
