# Supplementary figures and images for: Endosymbiosis in trypanosomatid protozoa: the bacterium division is controlled during the host cell cycle
Source: Front Microbiol. 2015 Jun 2;6:520. doi: 10.3389/fmicb.2015.00520 (PMC4451579; doi:10.3389/fmicb.2015.00520)

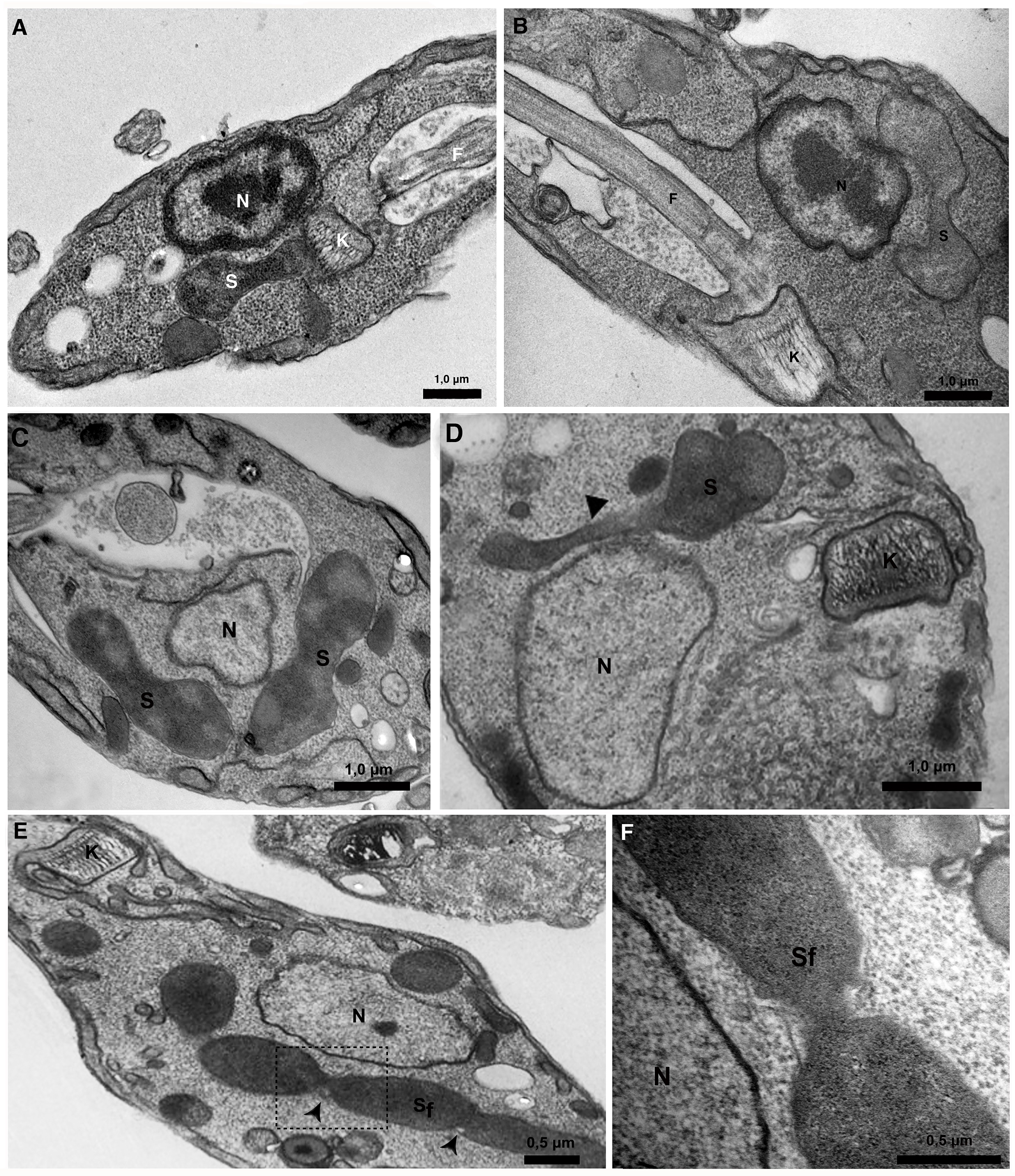

Supplement: Supplementary file 1 [file Image1.TIF]

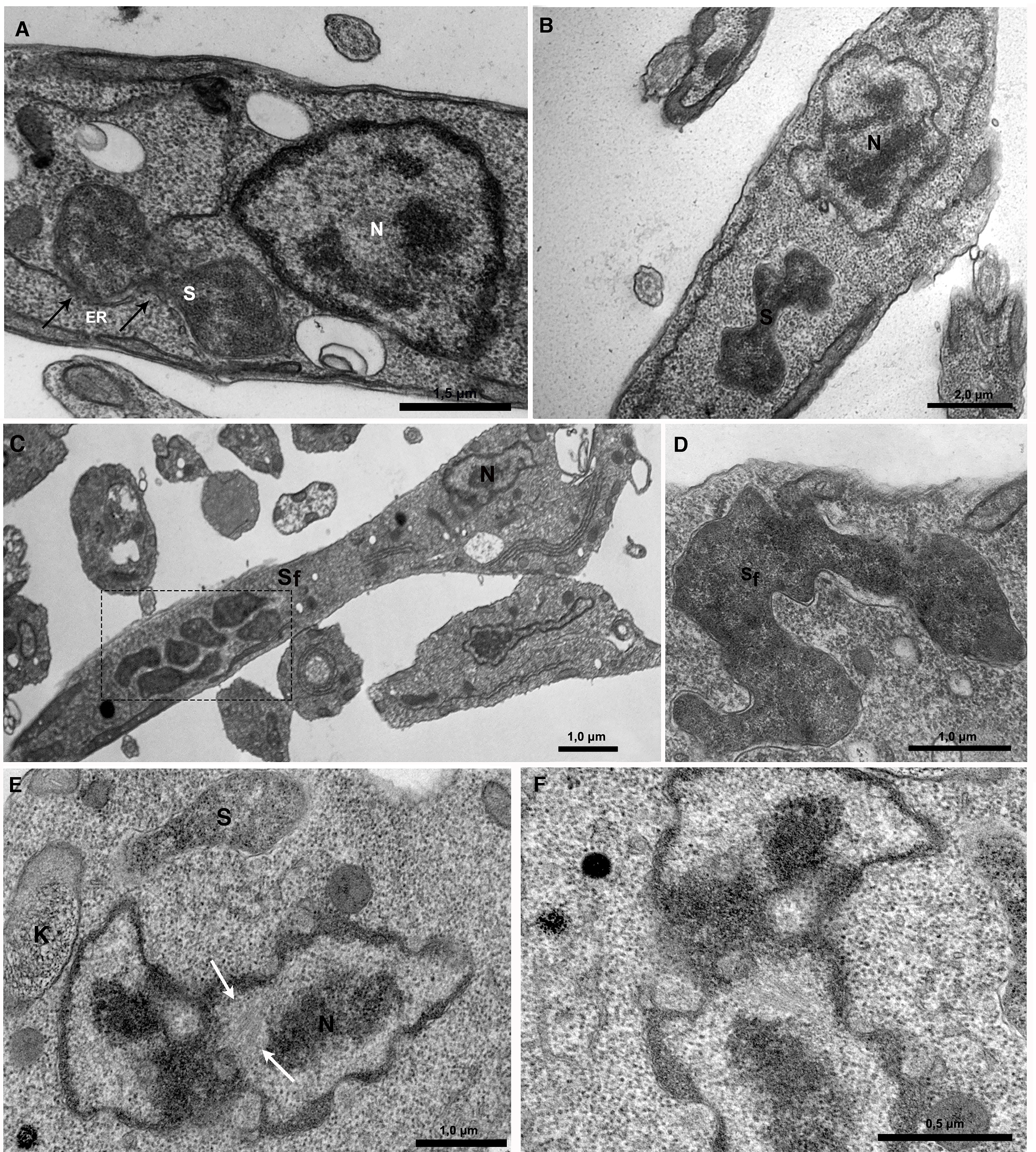

Supplement: Supplementary file 2 [file Image2.TIF]

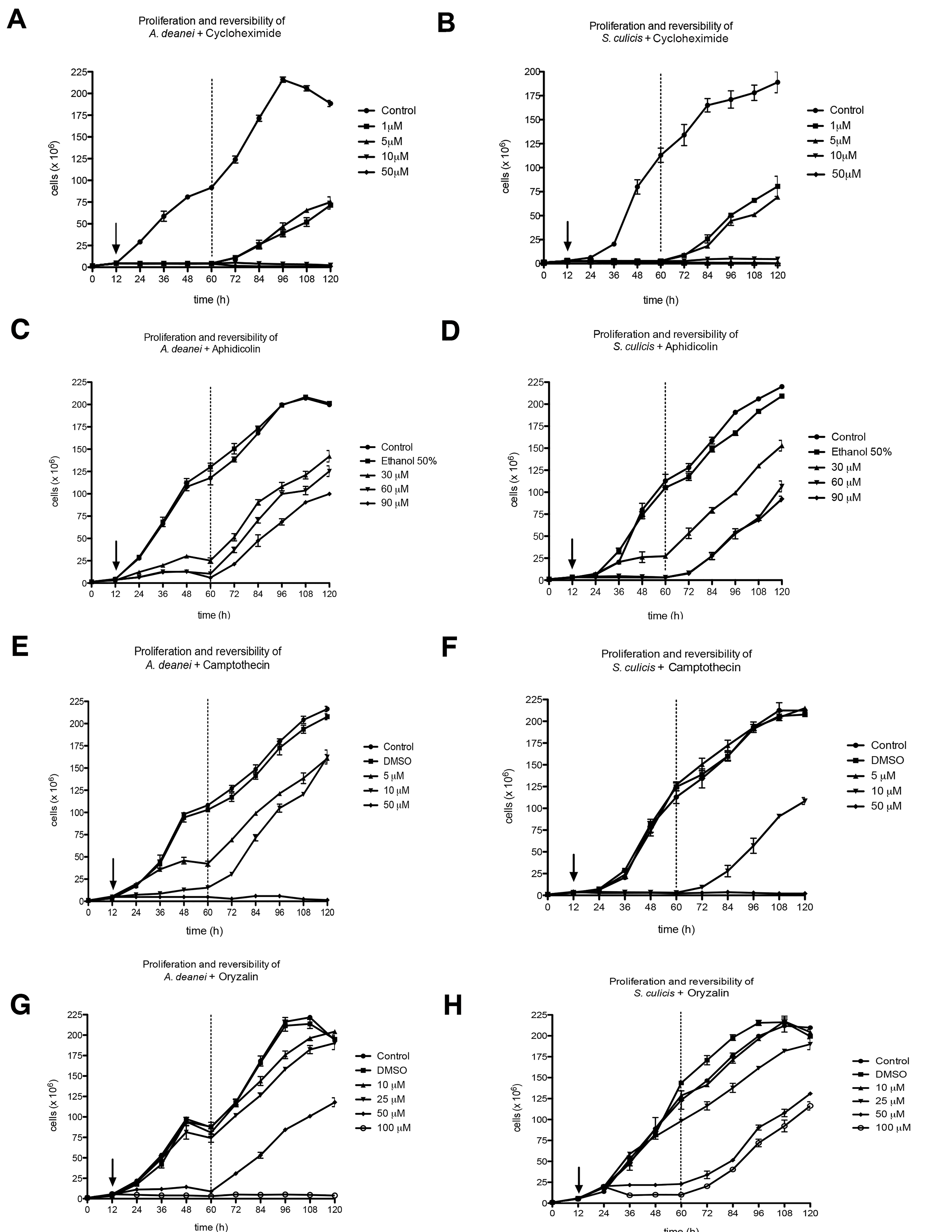

Supplement: Supplementary file 3 [file Image3.TIF]

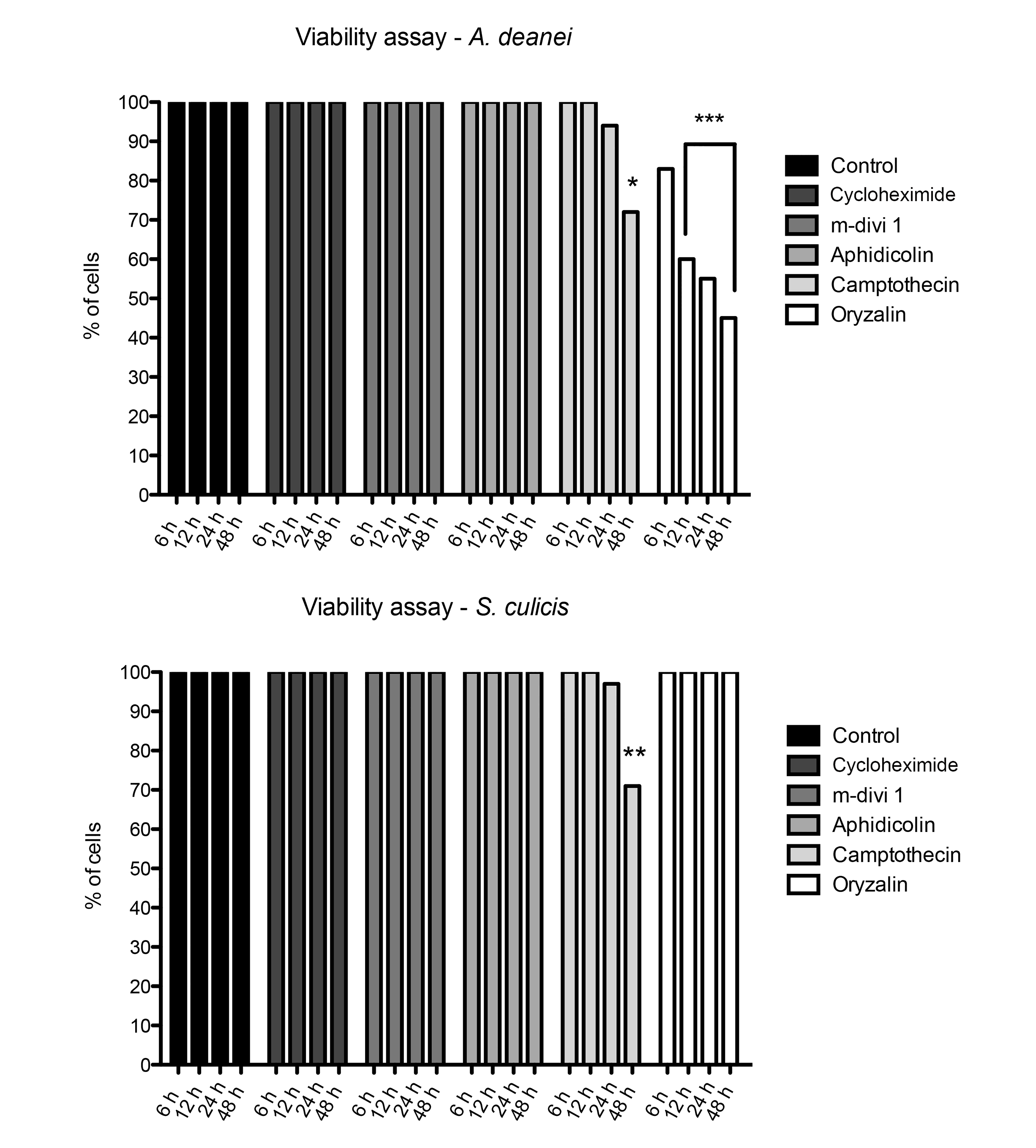

Supplement: Supplementary file 4 [file Image4.TIF]

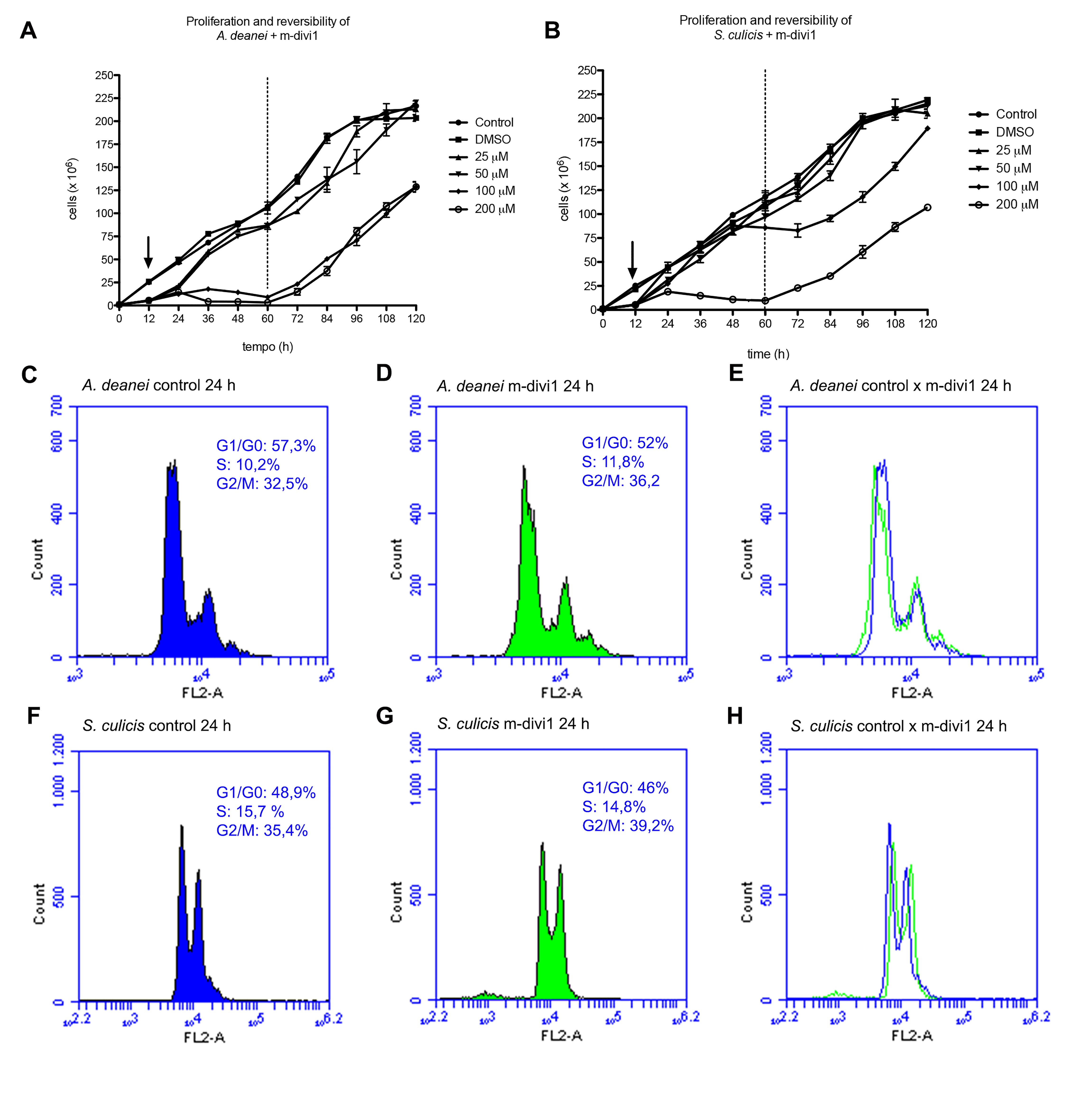

Supplement: Supplementary file 5 [file Image5.TIF]

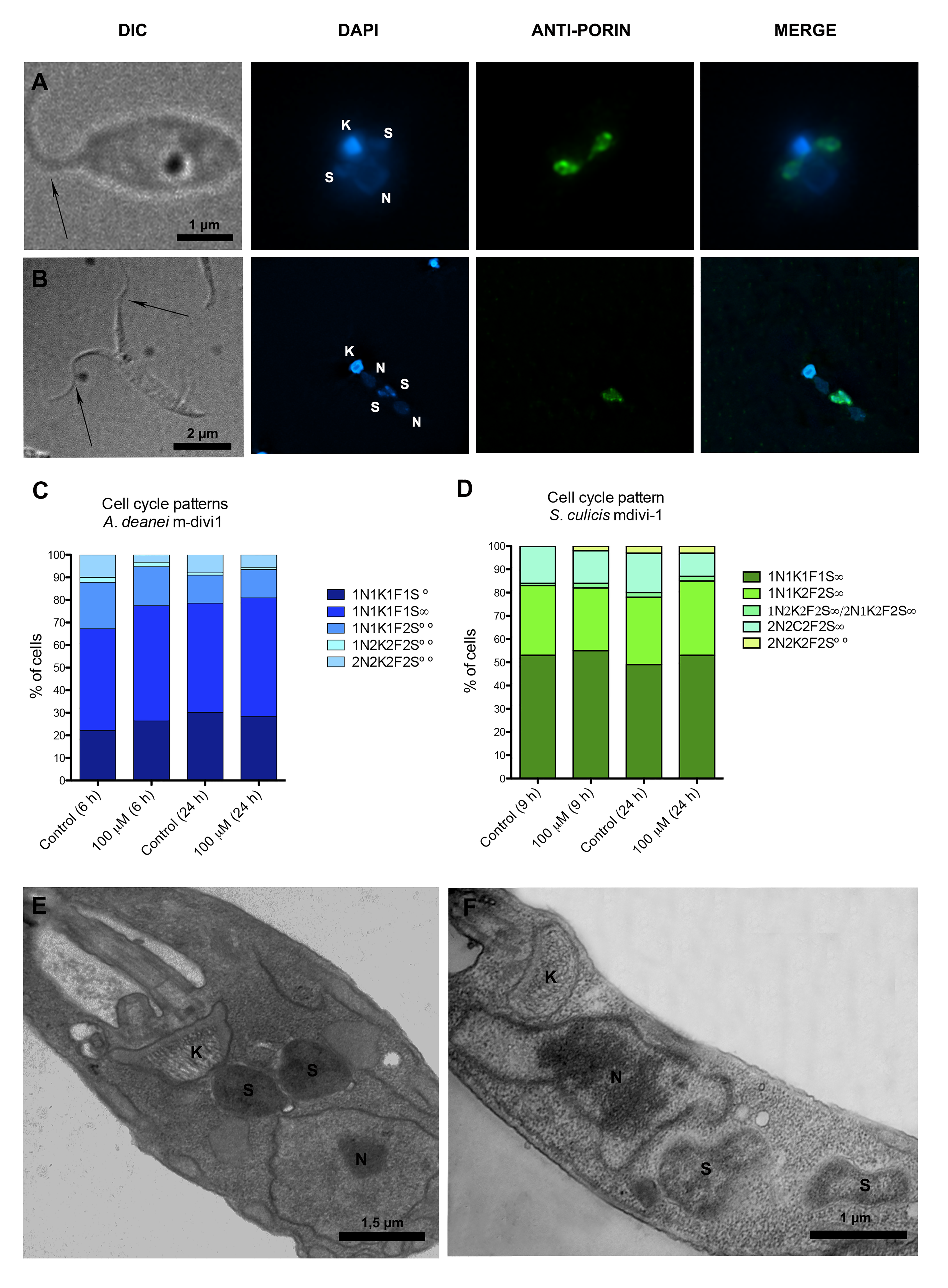

Supplement: Supplementary file 6 [file Image6.TIF]
